# Editorial: Immune Associated Mental Illnesses in Adolescents and Young Adults: Pathophysiological Role and Therapeutic Perspectives

**DOI:** 10.3389/fpsyt.2022.871719

**Published:** 2022-03-14

**Authors:** Richard B. Banati, Cathrin Rohleder, F. Markus Leweke, Norbert Mueller, Akira Sawa, Robert H. Yolken, Ian B. Hickie

**Affiliations:** ^1^Australian Nuclear Science and Technology Organisation, Sydney, NSW, Australia; ^2^Brain and Mind Centre, Translational Research Collective, Faculty of Medicine and Health, The University of Sydney, Sydney, NSW, Australia; ^3^Central Institute of Mental Health, Department of Psychiatry and Psychiatry, Medical Faculty Mannheim, Heidelberg University, Mannheim, Germany; ^4^Department of Psychiatry and Psychotherapy, Ludwig-Maximilians-University Munich, Munich, Germany; ^5^Solomon H. Snyder Department of Neuroscience, Johns Hopkins University School of Medicine, Baltimore, MD, United States; ^6^Department of Psychiatry and Behavioral Science, Johns Hopkins University School of Medicine, Baltimore, MD, United States; ^7^Department of Mental Health, Johns Hopkins University Bloomberg School of Public Health, Baltimore, MD, United States; ^8^Stanley Division of Developmental Neurovirology, Johns Hopkins School of Medicine, The Johns Hopkins Hospital, Baltimore, MD, United States

**Keywords:** autoimmune, immune-mediated, atypical presentation, depression, psychosis, encephalitis, immune markers, screening

The immune system's role in mental health has been discussed for decades, and immunological responses have been reported for many mental health issues. With the paradigm shift in psychiatry toward person-centered Precision Psychiatry (1), immune-mediated processes, too, are becoming more relevant as the current syndrome-based diagnostic concept fails to provide the necessary guidance in complex and/or treatment-resistant conditions.

Immune and autoimmune processes appear to be particularly critical at early disease stages in childhood, adolescents, and early adulthood. Following a possibly life-long trajectory, innate and adaptive immune responses draw and re-draw the boundaries between the biological self and non-self and influence, so we content, many facets of what individuals experience as mental health.

More recently, guidelines for autoimmune-mediated mental health disorders have been developed (2–5). These conditions are highly likely to involve a far broader range of autoantibodies than the few generally screened for. The question, therefore, arises as to which panel of clinical measurements and examinations contribute most to the uncovering of latent autoimmune-mediated mental disorders.

Engen et al. suggested that NMDA receptor antibodies in plasma must be interpreted carefully in an individual's clinical context. In their study, the prevalence of plasma IgG NMDAR antibodies in adolescent early-onset psychosis was comparable to healthy controls (6.5 vs. 2.9%). The presence of antibodies was not associated with specific clinical or radiological symptoms features.

The results of Bien et al. underline the importance and high specificity of cerebral spinal fluid (CSF) analysis for the reliable detection of neural antibodies. Paired serum and CSF samples from individuals with first-episode schizophrenic psychosis, clinical high risk for psychosis, and healthy volunteers were screened for eight different neural antibodies. Following comprehensive psychiatric and cognitive assessment, negative cerebral MRI, EEG, extended blood/CSF pathology, and physical and neurological examinations, all CSF samples were found to be autoantibody-negative, while three serum samples showed low-titer CASPR2 IgG antibodies and non-IgG NMDAR antibodies. However, CASPR2 IgG antibodies were below the laboratory cut-off for positivity, and non-IgG antibodies were not considered clinically relevant. Therefore, no cases of autoimmune encephalitis were identified in this cohort.

Moldavski et al. draw attention to the presence of atypical variations in the patterns of psychiatric manifestations in an individual with anti-NMDAR encephalitis who presented with a severe major depressive syndrome without any neurological signs or symptoms but unusually rapid onset, and reminiscent of potential early warning signs of clinical high risk for psychosis, and in need for a high dose of lorazepam.

Compared to the various forms of encephalitis seen in adults, encephalitis in children shows characteristic differences. Burrell et al. reported on the prospective Australian Childhood Encephalitis Study that documents altered behavior, particularly irritability/agitation, as a hallmark of childhood encephalitis. Disorientation/confusion and altered speech were also frequently observed, while psychotic features, such as hallucinations and paranoia, which are more common in adults, were rare in children. Furthermore, the absence of fever and a longer duration of altered behavior appeared to be associated with immune-mediated encephalitis.

To optimize care, psychiatrists and neurologists need to be familiar with differences in clinical presentations of children, adolescents, and adults with autoimmune encephalitis or primary psychiatric disorders. This aspect is discussed in the perspective article by Mooneyham et al. presenting current developments in assessing and treating children and adolescents with acute-phase autoimmune encephalitis. The article compares assessment and treatment models used in the United States, Canada, and Europe and concludes that inter-institutional and multidisciplinary research initiatives are needed to provide the best care to young people with autoimmune encephalitis and their supportive others.

Bahmani et al. investigated mental well being in multiple sclerosis, a chronic inflammatory autoimmune CNS disease, causing various signs and symptoms, including impaired vision, motor function, sensory perception, fatigue, sleep disturbances, and cognitive decline. In naturalistic observation, they found sleep patterns, physical activity, and psychological well being affected at an individual and fine-grained level 13.5 months after discharge from rehabilitation. On-trend, depression symptoms remained stable, physical activity levels decreased, and fatigue and subjective sleep scores worsened, while objectively measured wake time after sleep onset improved significantly.

While six articles in our collection examined the link between the immune system and the presence of psychiatric features or behavioral and cognitive changes, Ouyang et al. investigated the effects of antipsychotic treatment on cell-free mitochondrial DNA (cf-mtDNA) — a marker for oxidative stress and immune homeostasis — in individuals with first-episode schizophrenia. Before treatment, plasma cf-mtDNA levels in individuals with schizophrenia did not differ from those of healthy controls. Antipsychotic treatment reduced the cf-mtDNA levels, which correlated significantly with changes in the severity of positive and general schizophrenia symptoms. Although the reasons for the differences in cf-mtDNA remain to be explored, the authors hypothesize that cf-mtDNA may help assess clinical courses in the future.

Research into the immune system's role in psychiatric syndromes is progressing and promises more precise diagnosis and treatment of complex and/or treatment-resistant conditions.

The present collection of articles highlights the need to pay attention to individuals with atypical psychotic and mood syndromes. Atypical presentations include not only “odd” phenotypes with polymorphic psychiatric symptoms, significant cognitive involvement, sleep disturbances, and/or neurological phenomena (e.g., seizures), but also “unusual” responses to conventional treatment (e.g., need of unusually high doses, more motor side-effects). To recognize atypical presentations, it is important to appreciate the limits in the construct-validity syndrome-based diagnostic categories and instead apply standardized and complete sets of diagnostic procedures along with an extended history of previous medications, which may mask the presence of neurological symptoms (e.g., epileptiform activity in the EEG).

As the prevalence of autoimmune-mediated psychiatric syndromes appears low, at least by current definitions, we propose the systematic use of a more comprehensive screening that includes a broader range of specific and non-specific immune markers to advance our understanding of immune-mediated mental health conditions and enable faster identification and meaningful treatments for affected individuals. Systematic collection of individually highly variable immunological phenotypes and added to lifetime outcomes data is important in moving from ideal type-based diagnoses to precision psychiatry that individualizes therapeutic intervention to the needs rather than the diagnostic classification.

## Author Contributions

This Research Topic on Immune Associated Mental Illnesses in Adolescents and Young Adults was initially proposed and established by RBB, CR, FML, and IBH. All editors contributed to the management of our Research Topic. This editorial introduction was led by RBB, CR, FML, and IBH and reviewed and revised by the entire editorial team. All authors contributed to the article and approved the submitted version.

## Conflict of Interest

FML is a shareholder of curantis UG (Ltd.) and CR is a shareholder of lero bioscience UG (Ltd.). IBH was an inaugural Commissioner on Australia's National Mental Health Commission (2012–18). He is the Co-Director, Health, and Policy at the Brain and Mind Centre (BMC) University of Sydney, Australia. The BMC operates an early-intervention youth services at Camperdown under contract to headspace. IBH has previously led community-based and pharmaceutical industry-supported (Wyeth, Eli Lily, Servier, Pfizer, AstraZeneca) projects focused on the identification and better management of anxiety and depression. He was a member of the Medical Advisory Panel for Medibank Private until October 2017, a Board Member of Psychosis Australia Trust, and a member of Veterans Mental Health Clinical Reference group. He is the Chief Scientific Advisor to, and a 5% equity shareholder in, InnoWell Pty Ltd. InnoWell was formed by the University of Sydney (45% equity) and PwC (Australia; 45% equity) to deliver the $30 M Australian Government-funded Project Synergy (2017–20; a 3-year program for the transformation of mental health services) and to lead transformation of mental health services internationally through the use of innovative technologies. The remaining authors declare that the research was conducted in the absence of any commercial or financial relationships that could be construed as a potential conflict of interest.

## Publisher's Note

All claims expressed in this article are solely those of the authors and do not necessarily represent those of their affiliated organizations, or those of the publisher, the editors and the reviewers. Any product that may be evaluated in this article, or claim that may be made by its manufacturer, is not guaranteed or endorsed by the publisher.
